# Premaxillary Fat Pads on Magnetic Resonance Imaging (MRI) as a Marker of Steroid Side Effects

**DOI:** 10.7759/cureus.96041

**Published:** 2025-11-03

**Authors:** Roshwanth A, Tanvi Goel, Priya Garg, Sameer Peer, Arvinder Wander

**Affiliations:** 1 Pediatrics, All India Institute of Medical Sciences (AIIMS) Bathinda, Bathinda, IND; 2 Radiology, All India Institute of Medical Sciences (AIIMS) Bathinda, Bathinda, IND

**Keywords:** lipodystrophy, pediatric neuroinflammatory disorders, premaxillary fat pad hypertrophy, soft tissue changes, steroid-induced toxicity

## Abstract

Premaxillary fat pads visible on magnetic resonance imaging (MRI) are emerging as a sensitive marker for steroid side effects in chronic diseases including pediatric neuroinflammatory disorders where steroids offer proven efficacy in inflammation control and long-term steroids are the mainstay of treatment. However, their use carries significant risks of systemic toxicity, often presenting as abnormal fat redistribution or growth suppression, which can be challenging to identify clinically, especially in children who are overweight or have subtle physical changes. Through this case report, we highlight that routine follow-up MRI in pediatric neuroinflammatory disorders can serve a dual purpose-not only monitoring disease resolution but also acting as a sensitive tool for the early detection of premaxillary fat pad hypertrophy as an indicator of steroid toxicity. Leveraging MRI’s superior soft tissue contrast and decreased radiation exposure, such assessments enable objective and timely quantification of steroid-induced changes, facilitating prompt diagnosis and optimal management, particularly in cases where clinical features are subtle or masked.

## Introduction

Premaxillary fat pads visible on magnetic resonance imaging (MRI) are emerging as a sensitive marker for steroid side effects in chronic diseases including pediatric neuroinflammatory disorders where steroids offer proven efficacy in inflammation control and long-term steroids are the mainstay of treatment. However, their use carries significant risks of systemic toxicity, often presenting as abnormal fat redistribution or growth suppression, which can be challenging to identify clinically, especially in children who are overweight or have subtle physical changes. Literature advocates regular monitoring, ideally using imaging and anthropometric measures alongside biochemical tests to catch these changes early and prevent long-term morbidity [1]. MRI is increasingly recognized for its role in objectively quantifying steroid-induced soft tissue changes. Imaging of epicardial and pericardial fat pads via cardiac MRI has proven the ability to distinguish high-dose steroid users from steroid-naïve controls, emphasizing the degree to which steroids alter fat distribution systemically [2]. Through this case report, we highlight that routine follow-up MRI in pediatric neuroinflammatory disorders can serve a dual purpose-not only monitoring disease resolution but also acting as a sensitive tool for the early detection of premaxillary fat pad hypertrophy as an indicator of steroid toxicity. Leveraging MRI’s superior soft tissue contrast and decreased radiation exposure, such assessments enable objective and timely quantification of steroid-induced changes, facilitating prompt diagnosis and optimal management, particularly in cases where clinical features are subtle or masked.

## Case presentation

**Figure 1:**
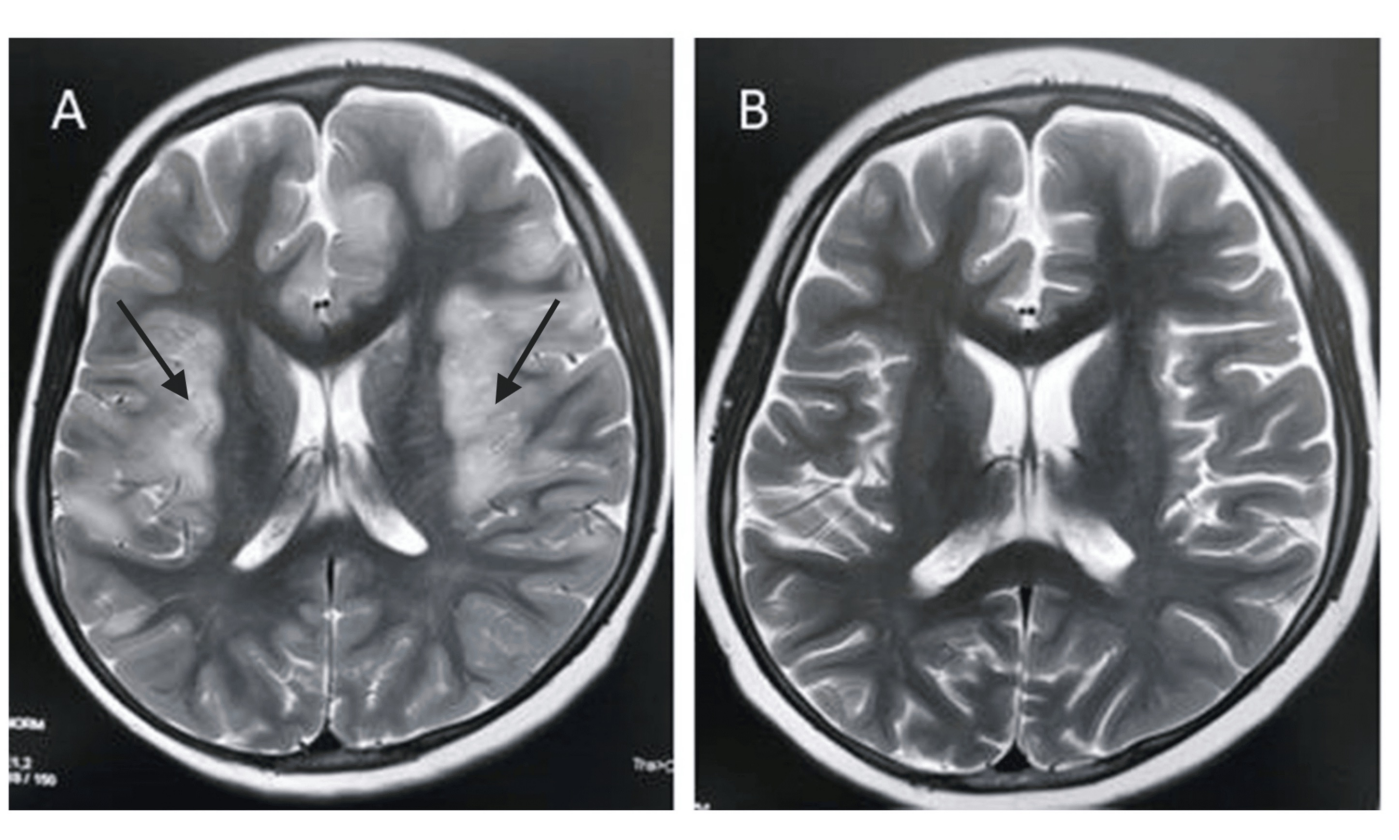
MRI brain showing the primary lesion and its resolution on follow-up (A) T2W axial image of the brain at the level of the lateral ventricles shows symmetric patchy juxta-cortical hyperintense signal involving the bilateral peri-insular white matter (black arrow); (B) T2W axial image corresponding to A, at the 6-week follow-up, shows complete resolution of the signal changes after steroid therapy. MRI: magnetic resonance imaging: T2W: T2-weighted

A 10-year-old boy with no prior medical history presented with altered consciousness, weakness in lower limbs, urinary retention, and decreased vision following a febrile illness. Neurological evaluation showed intact cranial nerve and brain stem functions, lower limb weakness, and exaggerated deep tendon reflexes (DTRs) at the knees and ankles. No significant findings were noted in his family. CSF examination revealed pleocytosis (5 WBCs/mm³) and increased protein (60 mg/dL). MRI brain at the level of the lateral ventricles shows symmetric patchy juxta-cortical hyperintense signal involving the bilateral peri-insular white matter (Figure 1). Myelin oligodendrocyte glycoprotein (MOG) antibodies with a titer of 1:160 were found in serum, which was suggestive of MOG-associated disease (MOGAD). The initial treatment plan included intravenous methylprednisolone at 30 mg/kg/day for five days, followed by oral corticosteroids at a full dose (2 mg/kg/day) for six weeks, after which the steroids were planned to be tapered over the next six weeks. One month after the initiation of steroid therapy, the child experienced new-onset behavioral changes, anxiety, headaches, and sleep disturbance, but there were no visual symptoms or overt facial swelling. These neuropsychiatric symptoms were initially attributed to the underlying disease rather than steroid medication and were managed conservatively.

**Figure 2:**
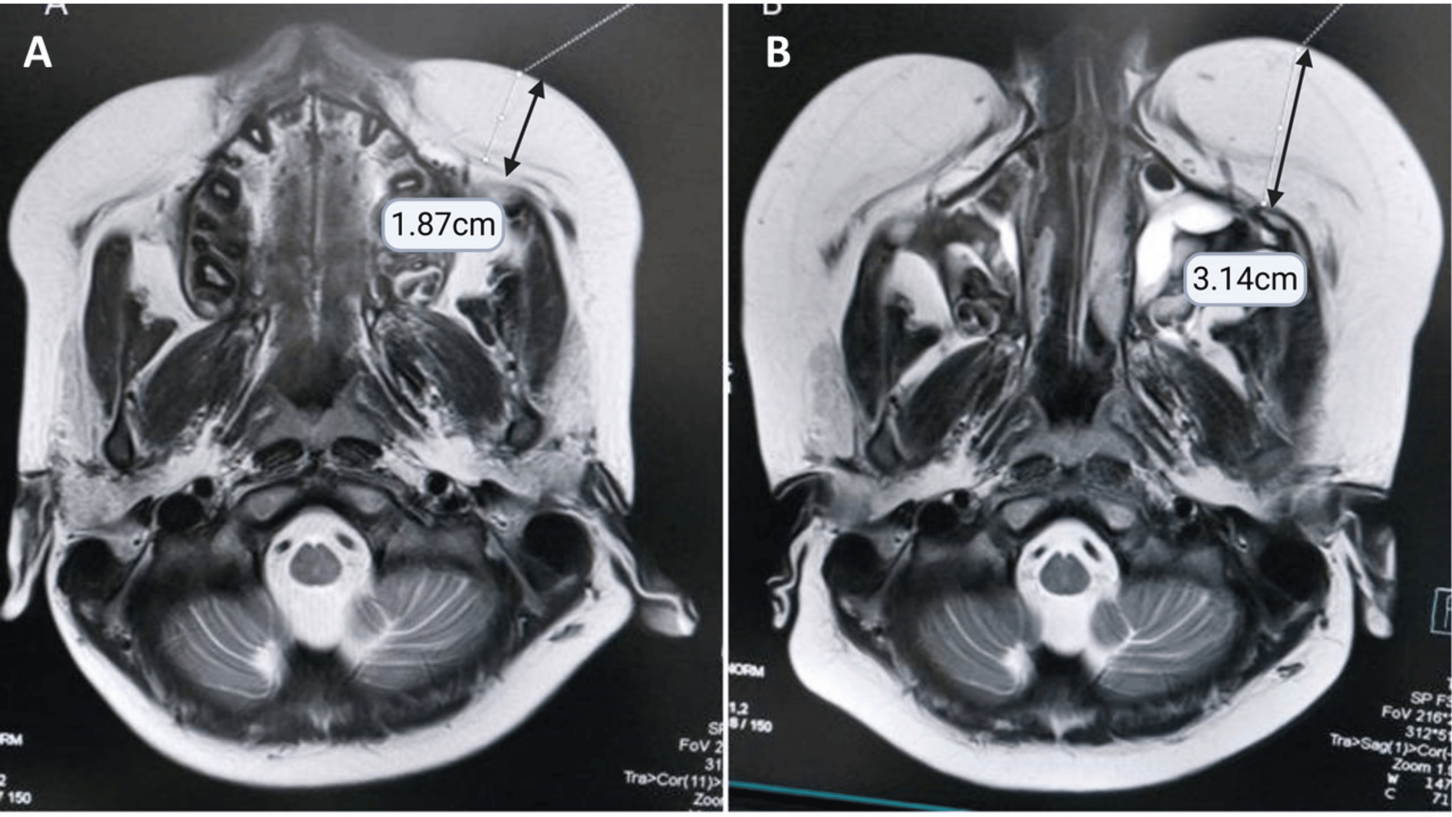
MRI brain showing premaxillary fat hypertrophy (A) T2W axial image of the face at the time of clinical presentation shows premaxillary fat thickness of 18 mm in the left maxillary region. (B) T2W axial image of the face at follow-up after 6 weeks of steroid therapy, at the same level corresponding to C, shows significant hypertrophy of the premaxillary fat, which measures at 32 mm. T2W: T2-weighted; MRI: magnetic resonance imaging

**Figure 3:**
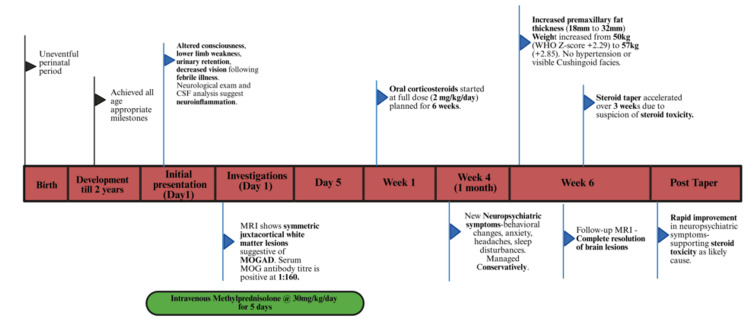
Timeline of events in our case CSF: cerebrospinal fluid; MRI: magnetic resonance imaging; MOG: myelin oligodendrocyte glycoprotein; MOGAD: myelin oligodendrocyte glycoprotein-associated disease

At the six-week follow-up, MRI brain-conducted to monitor the resolution of neuroinflammatory lesions-revealed complete lesion resolution (Figure 1) but a substantial increase in premaxillary fat thickness, from 18 to 32 mm (Figure 2). During this period, the child’s weight increased from 50 kg (WHO Z-score: +2.29) to 57 kg (+2.85), though blood pressure remained normal with no visible Cushingoid facies noted, possibly masked by the child’s pre-existing overweight status. Upon identification of the increase in premaxillary fat on MRI, a decision was made to accelerate the steroid tapering, which was tapered over three weeks. This led to rapid improvement in symptoms, further supporting the suspicion of evolving steroid toxicity that was otherwise subclinical and not clinically obvious due to pre-existing obesity (Figure 3; Table 1).

**Table 1:** Serial clinical and neuroimaging features of our case IV: intravenous; MRI: magnetic resonance imaging

Time point	Fat thickness (mm)	Weight (kg)	WHO weight Z-score	Blood pressure	Symptoms	MRI findings
Admission (Day 0)	Not measured	50	+2.29	Normal (50th to 90th centile)	Altered consciousness, limb weakness, urinary retention, vision decrease	Bilateral peri-insular white matter hyperintensities
End of IV steroids (Day 5)	Not measured	50		Normal (50th to 90th centile)	Neurological improvement	Same lesions, pretreatment MRI
1 month (Day 30)	18	54		Normal (50th to 90th centile)	New anxiety, behavioral changes, headaches	Partial lesion resolution
6 weeks (Day 42)	32	57	+2.85	Normal (50th to 90th centile)	Neuropsychiatric symptoms, no Cushingoid signs	Complete lesion resolution, increased fat thickness
6-9 weeks (Days 42-63)	Monitoring ongoing	58		Normal (50th to 90th centile	Symptom improvement post-taper	No new lesions

## Discussion

Corticosteroids are cornerstone therapies for a range of pediatric neuroinflammatory disorders, which include acute demyelinating syndromes (ADS), including acute disseminated encephalomyelitis (ADEM), neuromyelitis optica spectrum disorder (NMO-SD), optic neuritis (ON), MOGAD, and CNS infections with immune-mediated inflammation [3]. While effective in controlling disease, their systemic side effects-particularly steroid-induced toxicity-can be insidious and often masked by underlying comorbidities or the primary disease process itself. This case highlights the importance of careful toxicity monitoring, especially since clinical manifestations may be subtle or atypical in overweight children.

Long-term or high-dose steroid therapy commonly leads to abnormal fat redistribution (lipodystrophy), including both lipohypertrophy (localized fat accumulation) and lipoatrophy (fat loss) [4]. Hypercortisolism is associated with characteristic fat accumulation in various body regions, including centripetal obesity with increased panniculus adiposus, omental, mesenteric, and perirenal fat, as well as distinctive features such as moon facies, dorsocervical “buffalo hump,” supraclavicular and epipericardial fat pads, dewlap fat deposits, and superior mediastinal widening [5]. Other features of steroid toxicity include growth suppression, hypertension, osteoporosis, fractures, immunosuppression, cataracts, and glaucoma [6]. Classic Cushingoid features such as facial rounding or dorsocervical fat pad enlargement may not be readily apparent in children who are already overweight or obese, compounding the diagnostic challenge. In this context, imaging studies-especially MRI-play a pivotal role. MRI’s high soft tissue contrast allows for sensitive detection of changes in soft tissue composition, particularly the premaxillary (buccal) fat pad, an area prone to steroid-induced hypertrophy [7].

The buccal fat pad represents a distinct anatomical fat compartment within the face that may undergo hypertrophy following corticosteroid exposure, attributable to the characteristic redistribution of adipose tissue associated with steroid metabolism [8]. Identification of specific imaging patterns of fat pad enlargement or lipoatrophy can aid in the diagnosis of corticosteroid-related adverse effects. Radiological evaluation aids in distinguishing steroid-induced fat distribution changes from alternative pathologies, thereby supporting clinical decision-making and optimizing patient management [4]. In the current case, a serial MRI done for primary disease monitoring inadvertently provided early evidence of steroid toxicity by demonstrating significant buccal fat pad hypertrophy even before clinical Cushingoid features were evident. This underscores the need for radiologists and clinicians to actively assess fat pad measurements during follow-up MRI studies in children on corticosteroids, not as a separate diagnostic pursuit, but as an adjunct to standard monitoring for disease resolution.

While MRI-based quantification of premaxillary fat hypertrophy offers high sensitivity for detecting early steroid-induced lipodystrophy, particularly when overt clinical signs are lacking, its specificity is moderate, as similar facial fat changes may occasionally occur with obesity or endocrinopathies. Emerging MRI-based volumetric and fat-fraction techniques have demonstrated superior sensitivity compared to CT or anthropometric indices, providing an objective measure of regional fat redistribution. Thus, integrating premaxillary fat pad evaluation with biochemical profiling offers a complementary approach, improving both specificity and sensitivity for early detection of steroid toxicity in pediatric patients. The patient’s pre-existing overweight status (WHO Z-score +2.29 at baseline) was acknowledged as a potential confounder that could mask classical Cushingoid features such as facial rounding. We accounted for this by closely monitoring fat pad thickness on neuroimaging and weight gain trends, which suggested evolving steroid toxicity despite the absence of typical features externally.

MRI confers advantages over computed tomography (CT) by avoiding radiation exposure and offering enhanced soft tissue resolution, which is particularly relevant in pediatric patients [9]. Improved MRI techniques in conjugation with biochemical markers such as hair cortisol analysis offer an integrated framework for evaluating steroid side effects both systemically and in target tissues including facial fat compartments [10]. Although clinical assessment and history of steroid exposure remain the cornerstone for diagnosing steroid toxicity, MRI-based detection of premaxillary fat hypertrophy can serve as an early diagnostic indicator, especially in challenging cases where physical findings are equivocal or masked. Steroid-sparing agents can also be used to minimize the long-term toxicity associated with chronic steroid therapy, especially during tapering phases in the management of chronic inflammatory and autoimmune conditions [11]. Their use is supported by robust evidence across multiple diseases, but it is crucial to understand both their benefits and potential risks.

Although steroid-sparing agents, such as biologic therapies including IL-1 and IL-6 inhibitors, are increasingly used in pediatric neuroinflammatory and hyperinflammatory disorders, their monitoring typically involves a combination of clinical assessments, laboratory biomarkers (e.g., CRP and IL-6), and advanced imaging modalities-such as molecular and imaging biomarkers like neurofilament light chain, exosomal tau, and inflammatory markers [12,13]. Currently, there is no established protocol for utilizing premaxillary fat thickness as a universal imaging marker across various neuroinflammatory etiologies.

A significant limitation in evaluating steroid-induced premaxillary fat pad hypertrophy in children is the absence of established normative MRI data stratified by age and sex. The marked interindividual variability in facial adiposity influenced by genetic, familial, and nutritional factors precludes reliance on absolute fat pad thickness values. Consequently, identifying relative interval changes on serial imaging remains the most practical and clinically relevant approach for detecting steroid toxicity. Future research should prioritize multicenter, prospective studies to systematically measure premaxillary fat pad dimensions in diverse pediatric populations, aiming to develop robust, standardized normative reference ranges.

## Conclusions

This report demonstrates that in the context of neuroinflammatory disease requiring long-term steroid therapy, routine MRI monitoring for primary disease resolution can concurrently yield valuable insights into early detection of steroid toxicity. Careful attention to subtle imaging changes, such as increases in buccal or premaxillary fat pads, can enable timely intervention by adjusting steroid dose or adding steroid-sparing agents and protect against more severe metabolic or cosmetic complications. As advances in imaging and quantitative fat measurement tools continue to evolve, MRI’s role as both a disease and toxicity monitor is poised to expand, offering improved outcomes and safer therapies for pediatric patients.
